# Combined bacterial and fungal intestinal microbiota analyses: Impact of storage conditions and DNA extraction protocols

**DOI:** 10.1371/journal.pone.0201174

**Published:** 2018-08-03

**Authors:** Cécile Angebault, Amine Ghozlane, Stevenn Volant, Françoise Botterel, Christophe d’Enfert, Marie-Elisabeth Bougnoux

**Affiliations:** 1 Unité de Parasitologie-Mycologie, Service de Microbiologie clinique, Hôpital Necker-Enfants-Malades, Assistance Publique des Hôpitaux de Paris (APHP), Paris, France; 2 Université Paris Descartes, Sorbonne Paris-Cité, Paris, France; 3 Unité de Parasitologie-Mycologie, Département de Virologie, Bactériologie-Hygiène, Mycologie-Parasitologie, Unité transversale du traitement des infections (VBHMP–UT2I), DHU-VIC, CHU Henri Mondor, Assistance Publique des Hôpitaux de Paris (APHP), Créteil, France; 4 EA Dynamyc 7380 UPEC, ENVA, Faculté de Médecine de Créteil, Créteil; 5 Institut Pasteur, Bioinformatics and Biostatistics Hub—C3BI—USR 3756 IP CNRS, Paris, France; 6 Institut Pasteur, INRA, Unité Biologie et Pathogénicité Fongiques, Département Mycologie, Paris, France; Institute of Microbiology, SWITZERLAND

## Abstract

**Background:**

The human intestinal microbiota contains a vast community of microorganisms increasingly studied using high-throughput DNA sequencing. Standardized protocols for storage and DNA extraction from fecal samples have been established mostly for bacterial microbiota analysis. Here, we investigated the impact of storage and DNA extraction on bacterial and fungal community structures detected concomitantly.

**Methods:**

Fecal samples from healthy adults were stored at -80°C as such or diluted in RNA*later*® and subjected to 2 extraction protocols with mechanical lysis: the Powersoil® MoBio kit or the International Human Microbiota Standard (IHMS) Protocol Q. Libraries of the 12 samples targeting the V3-V4 16S and the ITS1 regions were prepared using Metabiote® (Genoscreen) and sequenced on GS-FLX-454. Sequencing data were analysed using SHAMAN (http://shaman.pasteur.fr/). The bacterial and fungal microbiota were compared in terms of diversity and relative abundance.

**Results:**

We obtained 171869 and 199089 quality-controlled reads for 16S and ITS, respectively. All 16S reads were assigned to 41 bacterial genera; only 52% of ITS reads were assigned to 40 fungal genera/section. Rarefaction curves were satisfactory in 3/3 and 2/3 subjects for 16S and ITS, respectively. PCoA showed important inter-individual variability of intestinal microbiota largely overweighing the effect of storage or extraction. Storage in RNA*later*® impacted (downward trend) the relative abundances of 7/41 bacterial and 6/40 fungal taxa, while extraction impacted randomly 18/41 bacterial taxa and 1/40 fungal taxon.

**Conclusion:**

Our results showed that RNA*later*® moderately impacts bacterial or fungal community structures, while extraction significantly influences the bacterial composition. For combined bacterial and fungal intestinal microbiota analysis, immediate sample freezing should be preferred when feasible, but storage in RNA*later*® remains an option under unfavourable conditions or for concomitant metatranscriptomic analysis; and extraction should rely on protocols validated for bacterial analysis, such as IHMS Protocol Q, and including a powerful mechanical lysis, essential for fungal extraction.

## Introduction

The human intestinal microbiota is a highly dense ecosystem, containing a vast community of microorganisms living in intimate contact with our digestive system. Amongst those, bacteria are the most represented, but other inhabitants such as fungi, viruses or archaea are also part of the intestinal microbiota. Over the past decade, with the development of next-generation sequencing (NGS) platforms enabling high-throughput metagenomics, the human intestinal microbiota, and mostly its bacterial component, has been increasingly studied leading to advanced understandings of its role in health and disease [1–4]. A crucial step to obtain an unbiased and comparable representation of microbial communities using deep-sequencing techniques lays in the use of appropriate methods for specimen collection, storage and preparation prior NGS. Several authors have pointed out important variations regarding the yield and/or quality of isolated DNA associated with certain extraction protocols [5–9] and the community composition associated with storage conditions of fecal specimen [6,7,10–14] and/or extraction protocols [8,12,15–19]. In an effort to optimize the quality and comparability of data generated in human metagenomics research, the International Human Microbiota Consortium (IHMC) conducted a project (the International Human Microbiota Standards, IHMS) aiming at optimizing methods and proposing standard operating procedures (SOPs) to assess the intestinal microbiota with the utmost accuracy and comparability [12]. As most studies focused primarily on the bacterial component of the intestinal microbiota, the IHMS SOPs were designed specifically for optimal bacterial microbiota analysis. Two protocols were proposed by IHMC: the IHMS SOP Protocol Q (based on Qiagen-lysis QIAGEN QIAAmp DNA Stool kit) and the Protocol H (a non-kit-based protocol) [12]. In a recent paper, Costea *et al* compared 21 extraction protocols for bacterial metagenomic analysis [8]. Of all protocols tested, the IHMS SOP Protocol Q seemed to be the best for both its extraction quality (ensuring correct assessment of bacterial diversity) and its good reproducibility [8]. Lately, it has become increasingly evident that the less abundant component of the intestinal microbiota (i.e., “rare biosphere”, [20]), and particularly its fungal component, should be studied concomitantly with bacteria to better understand trans-kingdom interactions in the intestinal tract [21–25]. However, the methodological issues are even greater when it comes to the fungal microbiota (i.e. mycobiota) analysis, in particular because fungi are accountable for less than 0.1% of the genes residing in the intestinal microbiota [20]. Moreover, the structure of the fungal cell wall is highly complex, including a thick layer of chitin, (1–3)-beta-D-glucan, (1,6)-beta-glucans, lipids and peptides and sometimes a surface layer of melanin [26]. These physical characteristics prevent fungal cell walls from being easily lysed [27]. As stressed out by some authors [28,29], the DNA extraction is a key issue when designing a study for fungal microbiota analysis. A variety of protocols have been used to recover fungal DNA from different samples (including feces), with some fungal-specific steps (bead-beating or enzymatic lysis) [30–36]. However, and in contrast with bacterial microbiota analysis, very few studies have addressed concretely these methodological issues for fungal intestinal microbiota purpose [5,16,37,38], and to our knowledge, none has discussed the question of the most appropriate protocols to perform an accurate assessment of combined bacterial and fungal intestinal microbiota analyses.

In the present study, we sought to investigate the impact (*i*) of two frequently-used feces storage conditions: the within two-hours freezing of the fecal sample without additive and the dilution of fecal sample in an acid nucleic stabilizer solution (RNA*later*®) prior freezing [13,14] and (*ii*) of two DNA extraction protocols: the above-mentioned validated IHMS SOP Protocol Q [8,12] and the frequently used PowerLyser PowerSoil® MoBio commercial kit [5,19], on bacterial and fungal community structures detected concomitantly in human fecal specimen.

## Methods

### Sample collection and storage conditions

**Fig 1:**
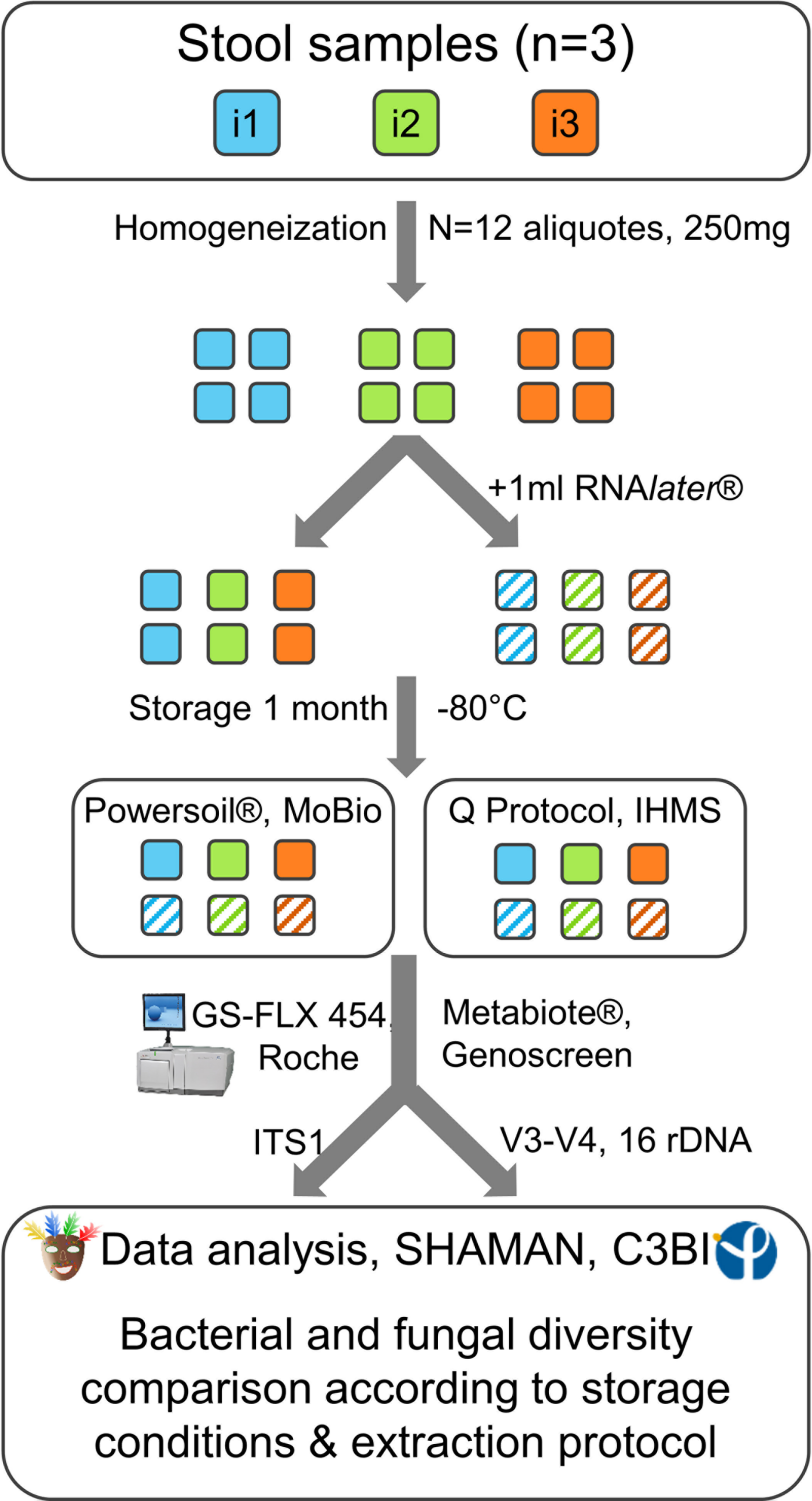
Workflow of the study. Comparison of the fungal and bacterial taxonomic diversity of fecal microbiota of 3 healthy individuals (i1, i2, i3) using 2 storage conditions (within two-hours freezing or RNA*later*® dilution before freezing) and 2 extraction protocols (IHMS Protocol Q and PowerSoil® MoBio kit).

Three healthy adults (i1, a 47 years-old man; i2 and i3, two women of 39 and 33 yrs-old, respectively), who had not received oral antibiotic or antifungal drugs for at least two months, volunteered to participate in the study and provided fresh fecal samples. Written informed consents were obtained from all volunteers in accordance with the Ethics Committee of the Necker-Enfants Malades Teaching Hospitals (2018-MEBA-8). Within 2 hours, the samples were homogenized and distributed in 250 (±10) mg aliquotes. For each sample, half of the aliquotes were diluted in 1 ml of RNA*later*® (Ambion, Inc., Austin, TX, USA) before storage at -80°C, while the other half were only frozen. All aliquotes were stored for one month at -80°C before being processed (Fig 1).

### DNA extraction protocols

For each sample and each storage condition, two different genomic DNA extraction protocols were used (PowerSoil® MoBio kit and IHMS Protocol Q, see below). All samples stored in RNA*later*® were subjected, prior extraction, to two rounds of centrifugation (10000g, 5 min)—PBS rinsing. Negative extraction controls (250 μL DNA-free water) were processed in the same way as samples.

#### MoBio PowerLyzer PowerSoil® DNA Isolation kit (QIAGEN, Carlsbad, USA)

The aliquotes were pipetted into PowerLyzer® 0.1 mm glass bead tubes and submitted to mechanical lysis, running two cycles of 30 secs at 6400 rpm on MagNA Lyser Instrument (Roche, Indianapolis, USA) with a 1 min rest on ice between cycles. We then proceeded as recommended by the manufacturer, with minor adjustments: after mechanical lysis, we centrifuged the Glass Bead Tubes (step 7 of the manufacturer’s protocol) for 5 mins instead of 30 secs and at the final centrifugation step (step 22 of manufacturer’s protocol), we waited for 5 minutes at room-temperature before spinning at 10000g for 1 min (instead of 30 secs).

#### Protocol Q, International Human Microbiota Standards [39]

This protocol, using QIAamp DNA stool kit (QIAGEN, Carlsbad, USA), has been recommended by the IHMC in the IHMS project (standard operating procedure 06 [39]) as a robust and reproducible DNA extraction method for fecal samples to be used for metagenomics analysis targeting intestinal bacterial microbiota. We proceeded with our aliquotes as recommended in the IHMS-SOP 06 [39] with only minor adjustments regarding mechanical lysis (step 1 and step 3 of IHMS-SOP 06): we used 0.1 mm glass beads instead of 0.1 mm zirconium beads and we performed 8 bead-beating cycles of 1 min at 6400 rpm on MagNA Lyser instead of FastPrep^TM^ Instrument with 5 minutes resting between cycles.

### PCR amplification and sequencing

Amplicon libraries, targeting the V3-V4 16S region for bacteria and the ITS1 region for fungi (primers ITS1F[40] and ITS2[41]), were prepared using the Metabiote® protocole (Genoscreen, Lille, France) and sequenced on GS-FLX 454 (Roche Life Sciences, Branford, USA) (Fig 1), resulting in 199631 and 206412 sequence reads of 388 nt and 249 nt (median length) respectively for 16S and ITS1. A positive qualitative control consisting in an artificial bacterial community (ABC, Metabiote®, Genoscreen), obtained from 11 different strains belonging to 11 genera (*Pseudomonas*, *Escherichia*, *Helicobacter*, *Neisseria*, *Clostridium*, *Streptococcus*, *Lactobacillus*, *Enterococcus*, *Bacillus*, *Propionibacterium*, *Actinomyces*) was used for V3-V4 16S amplification, while DNA from a *Candida albicans* strain was used for ITS1 amplification control (Metabiote®, Genoscreen). Reads with a positive match with human or phiX174 phage were removed. Library adapters, primer sequences, and base pairs occurring at 5′ and 3′ ends with a Phred quality score <20 were trimmed off by using Alientrimmer (v0.4.0) [42]. Resulting amplicons were clustered into operational taxonomic units (OTU) with VSEARCH [43]. The process includes several steps for dereplication, singletons removal, and chimera detection. The clustering was performed at 97% sequence identity threshold. The input amplicons were then mapped against the OTU set to get an OTU abundance table containing the number of reads associated with each OTU. The whole process, available on SHAMAN (http://shaman.pasteur.fr/ [44]) (section raw reads), led to retain 171869 and 199089 sequence reads (median length: 389 and 250 nt) clustering in 130 and 272 OTUs for 16S and ITS1 analyses, respectively (S1 Table). All reads are available on EBI ENA (Accession number PRJEB25216; https://www.ebi.ac.uk/ena).

### Taxonomic assignment, diversity and statistical analyses

The OTUs’ taxonomical annotation was performed using blast against Greengenes 13.5, Silva 128 and RDP classifier for 16S rDNA and UNITE [45], Targeted Host-associated Fungi (THF) version 3 (Underhill) [37], and Findley [46] for ITS1 analysis, applying criteria of ≥75% and ≥94.5% homology with reference sequences for annotation at phylum or genus level [47], respectively and <10^−5^ e-values. A complementary analysis of fungal OTUs non-assigned to the genus level with UNITE, THF or Findley database was performed using MycoBank [48].

The normalization of OTU counts was performed at the OTU level using DESeq2 normalization method [49]. The generalized linear model (GLM) implemented in the DESeq2 R package was then applied to detect differences in abundance of genera, at general or individual level, between the extraction condition (IHMS, MOBIO) and the RNA*later®* usage (yes, no). We defined a GLM that included the individual, the extraction condition and the RNA*later®* usage as main effects and an interaction between extraction condition and the RNA*later®* usage. This interaction was useful to model the pairing between successive measurements coming from the same individual. Resulting *P-*values were adjusted according to the Benjamini and Hochberg procedure.

We computed rarefaction curves to evaluate the quality of the deep-sequencing effort in regard of taxonomic diversity assessment. We performed principal coordinate analyses (PCoA), based on bray distance, to evaluate the between-sample dissimilarities and we computed different diversity indexes (Shannon, Simpson) to compare the homogeneity of the samples in terms of bacterial and fungal microbiota composition. The community structure and the relative abundances of the bacterial and fungal taxa were compared for each storage condition and extraction protocol. The results of the positive sequencing controls were satisfactory for 16S and ITS1: reads corresponding to the genera of the 11 bacterial strains of the ABC control and of *Candida* or the ITS1 control were detected.

## Results

### Influence of storage and extraction protocol on DNA quantity and quality

Beginning with the same fecal material mass in the 12 samples analyzed (3 individuals x 2 storage conditions x 2 extraction protocols), the IHMS extraction protocol seemed to produced higher rates of double-strand DNA (*P*-value = 0.01, average DNA yield 3.2 times higher (fold-range [1.2–5.1]) than the PowerSoil® MoBio kit (S1 Fig). Regarding the purity of the DNA extracted, we observed that the IHMS protocol produced higher rates of single-strand DNA; however, the 260/280 nm absorbance ratio (a metric of nucleic acids purity) were similar for both extraction protocols (*P*-value = 1, S1 Fig). We did not observe significant differences regarding DNA yield or purity according to storage condition.

### Bacterial diversity and community structure

**Fig 2:**
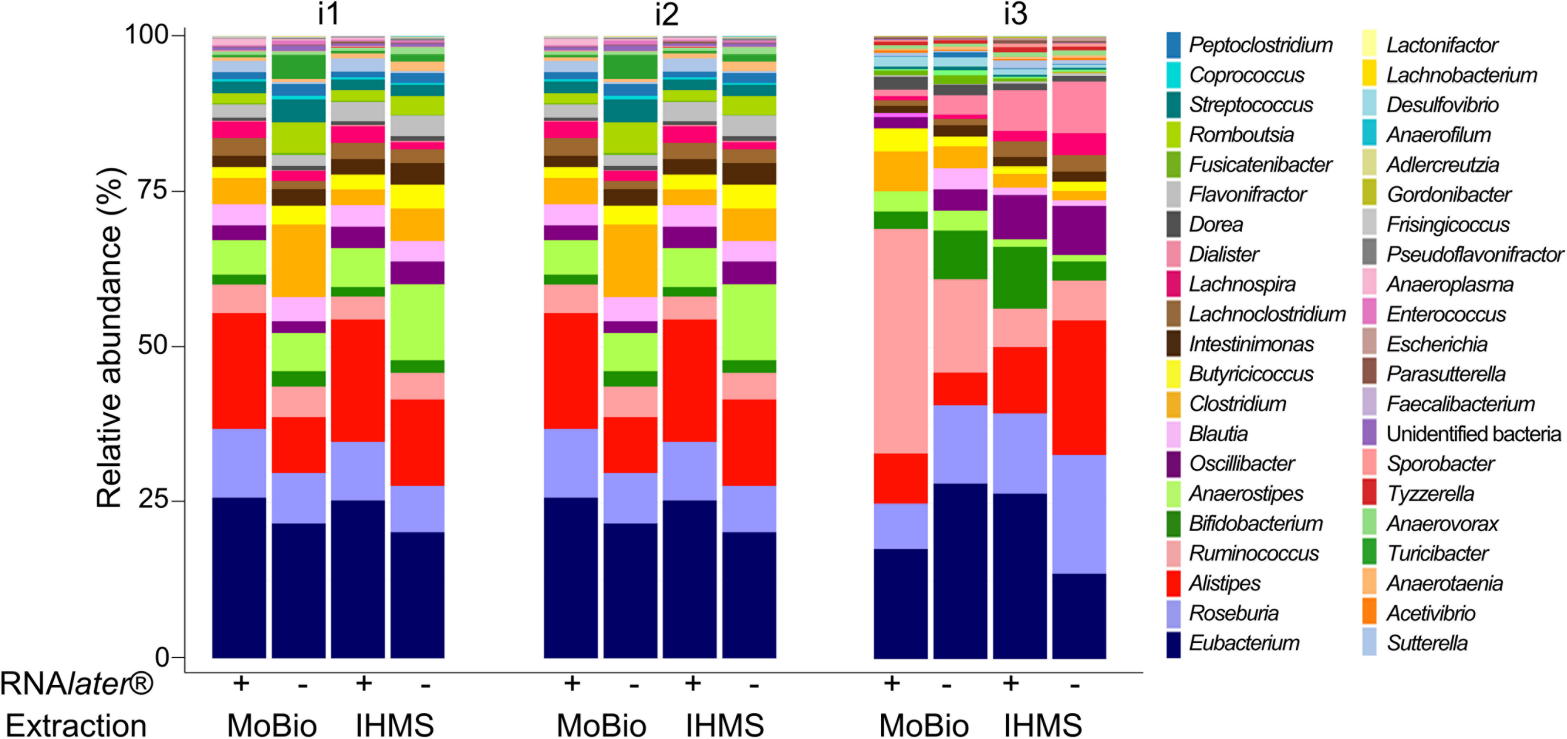
Relative abundance of taxa identified from faeces through 16S-sequencing according to storage or extraction conditions. Bacterial taxa were identified at genus level from fecal samples of 3 healthy individuals (i1, i2 and i3) using two storage conditions (without additive within two-hours freezing or RNA*later*® dilution before freezing) and two extraction protocols (IHMS Protocol Q and PowerSoil® MoBio kit). 16S rRNA gene ultra-deep-sequencing was performed using 454 technology.

Among the 171 869 reads analyzed for the V3-V4 16S target, 57188 were attributed to i1, 64803 to i2 and 49878 to i3 (S1 Table) with a mean number of 14322 ± 2357 per sample (range [10067–17927]). All reads, clustering into 130 bacterial OTU, were assigned as Bacteria: 69.0% of reads (70.2%, 70.7% et 65.3% respectively, for i1, i2 and i3) were assigned at species level; 30.8% at genus level (29.2, 29.3 and 34.6%, respectively) and 0.2% as unidentified bacteria (0.6, 0.1 and 0.1%, respectively) (S1 Table). Further analysis was conducted at genus level. The 130 bacterial OTUs gathered into 42 taxa: 41 at genus level and 1 taxon clustering unidentified bacteria. At individual level, we identified 39, 34 and 39 genera in i1, i2 and i3 microbiota, respectively. The rarefaction curves for i1, i2 and i3 samples reached a plateau, indicating that the bacterial diversity present in every sample had been satisfactorily detected (S2 Fig). Among the 41 taxa at genus level, 34 were common to all three individuals and 12 were “core” taxa, detected with a relative abundance over 1% in every sample (*Eubacterium*, *Roseburia*, *Alistipes*, *Ruminococcus*, *Bifidobacterium*, *Anaerostipes*, *Oscillibacter*, *Blautia*, *Clostridium*, *Butyricicoccus*, *Intestinimonas*, *Lachnoclostridium*; Fig 2). Using PCoA computed with bray distance, we observed that our 12 samples gathered predominantly according to individuals, rather than storage condition or extraction protocol (S3 Fig).

### Fungal diversity and community structure

**Fig 3:**
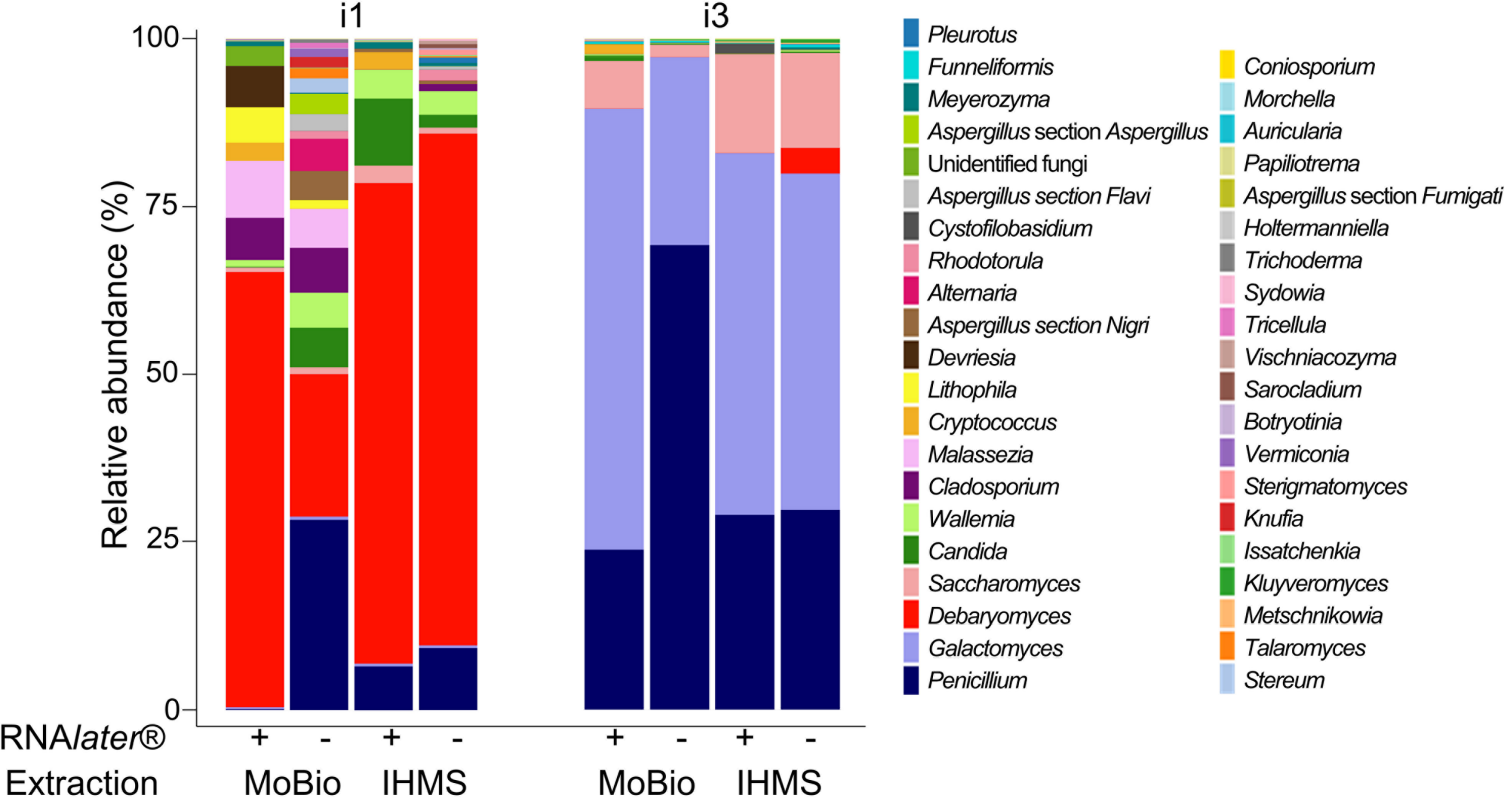
Relative abundance of taxa identified from faeces through ITS-sequencing according to storage or extraction conditions. Fungal taxa were identified at genus or section level from fecal samples of 2 healthy individuals (i1 and i3) using two storage conditions (without additive within two-hours freezing or RNA*later*® dilution before freezing) and two extraction protocols (IHMS Protocol Q and PowerSoil® MoBio kit). ITS1 ultra-deep sequencing was performed using 454 technology.

Of the 199089 reads analyzed for the ITS1 target, 65598 originate from the i1 samples, 62238 from i2 and 70253 from i3, with a mean number of 16591 ± 2584 reads per sample (range [12635–22374], S1 Table). The ITS1 reads gathered in 272 OTUs, of which 96 were assigned as Fungi (81 OTUS; 102478 [51.5%] reads) or Plantae (15 OTUs; 2434 [1.2%] reads) and 176 (94177 [47.3%] reads) were non assigned. At the individual level, the assignment rates varied greatly for i1, i2 and i3 (S1 Table). When 93.0% (n = 65332) of i3 reads were assigned as Fungi or Plantae, only 46.5% (n = 30989) and 13.8% (n = 8591) were assigned for i1 and i2, respectively (p-value<0.001). As only 65.3% (n = 66935) of overall reads assigned as Fungi (29.3%, 65.5% and 81.5% for i1, i2 and i3, respectively) were assigned at species level, further analysis was conducted at genus/section level. For i1 and i3, the rarefaction curves reached a plateau indicating that the fungal diversity was satisfactorily detected (S2 Fig). On the contrary, due to the important number of non-assigned reads (53647, 86.2%) for i2, the rarefaction curves were unsatisfactory and i2 samples were dismissed from further analysis. The fungal OTUs of i1 and i3 gathered into 41 taxa (37 at genus level, 3 at section level and 1 taxon clustering unidentified fungi). Among these taxa, 26 were common to both i1 and i3, but no “core” taxa (>1% relative abundance in every sample) were identified. However, the *Penicillium* taxon might be considered as a predominant taxon, as its relative abundance was over 1% in 7/8 samples. Comparing the profiles of i1 and i3, we observed highly different fungal community structures. When i1 harbored a diversified fungal microbiota, with 12 (/36) taxa having a relative abundance over 1% (*Debaryomyces*, 61.3%; *Penicillium*, 10.7%; *Candida*, 3.5%; *Wallemia*, 3.4%; *Cladosporium*, 3.4%; *Malassezia*, 3.3%; *Litophila*, 1.5%; *Devriesa*, 1.4%; *Aspergillus* section *Nigri*, 1.2%; *Saccharomyces*, 1.1%; *Cryptococcus*, 1% and *Alternaria*, 1%), i3 presented a low diverse profile, with only 3 (/30) very predominant taxa (*Galactomyces*, 45.6%; *Penicillium*; 43.5% and *Saccharomyces*, 8.1%) (Fig 3). Using PCoA computed with bray distance, we observed that our 8 samples analyzed gathered predominantly according to individuals, rather than storage condition or extraction protocol (S3 Fig).

### Comparison of bacterial and fungal diversity related to storage condition or extraction protocol

The bacterial community composition estimated through Shannon and Simpson indexes was comparable for the 12 samples processed (S2 Table). For the fungal community composition, Shannon and Simpson indexes were comparable in 7/8 samples analysed for i1 and i3. In one sample (i1; feces frozen without RNA*later*®; PowerSoil® MoBio kit), an increased diversity was found with all 40 fungal genera/section detected (Fig 3).

**Fig 4:**
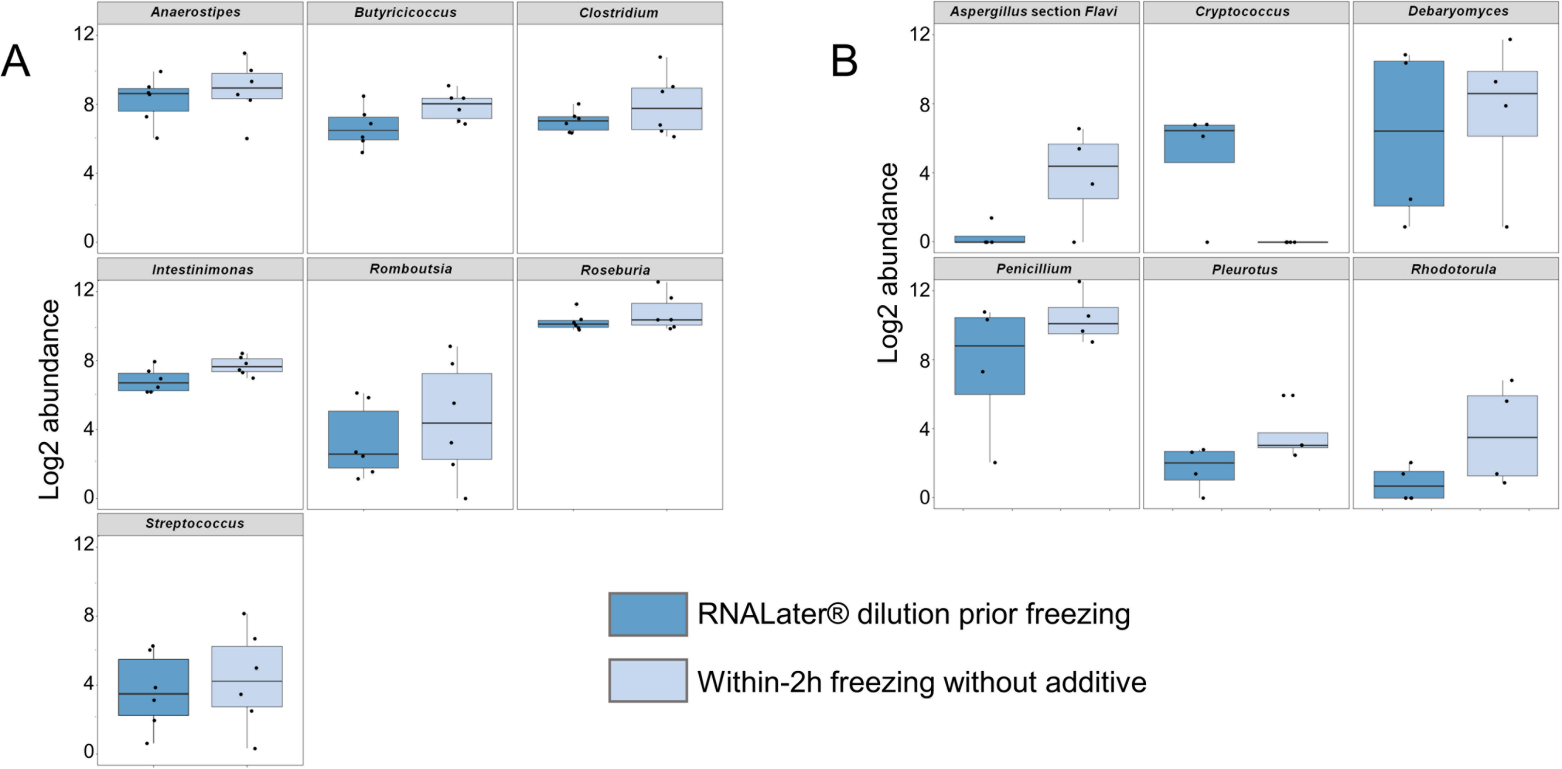
**Boxplot comparison of log2-abundance of bacterial (A) and fungal (B) taxa according to storage condition.** Bacterial diversity was assessed at genus level using 16S rRNA gene ultra-deep sequencing (454 technology) and fungal diversity at genus or section level using ITS1 ultra-deep sequencing. Boxplot of log2-abundance of taxa significantly different (*P*-value < 0,05) according to storage condition (RNA*later*® dilution before freezing [dark blue] vs. without additive within two-hours freezing [light blue]) during analysis at general level are presented.

The overall comparison of the relative abundances of the 41 bacterial genera and the 40 fungal genera or section between samples frozen immediately and samples frozen after dilution in RNA*later*® revealed significant statistical differences for 7/41 bacterial (*Anaerostipes*, *Butyricicoccus*, *Clostridium*, *Intestinimonas*, *Romboutsia*, *Roseburia*, *Streptococcus*) and 6/40 fungal taxa (*Aspergillus* section *Flavi*, *Cryptococcus*, *Debaryomyces*, *Penicillium*, *Pleurotus*, and *Rhodotorula*; Fig 4, S3 Table). Of note, 5/7 bacterial taxa were “core” taxa and 1/6 fungal taxon was the predominant *Penicillium* taxon. Further analysis at individual level revealed 5 more bacterial (*Enterococcus* and *Turicibacter* for i1; *Dialister* and *Peptoclostridium* for i3, and *Sutterella* for i1 and i3) and 1 more fungal (*Talaromyces* for i1) taxa significantly impacted by storage condition in terms of relative abundance (S4 Table, S4 Fig). Overall, we observed higher relative abundances in 11/12 and 6/7 of these bacterial or fungal taxa when samples were directly frozen compared to samples diluted in RNA*later*® prior freezing (Fig 4, S4 Fig). However, the taxonomic diversity was successfully assessed for bacteria and fungi whatever storage condition was used, except for one fungal taxon (*Cryptococcus*), which was detected only from samples stored with RNA*later*® (Fig 4, S4 Fig).

**Fig 5:**
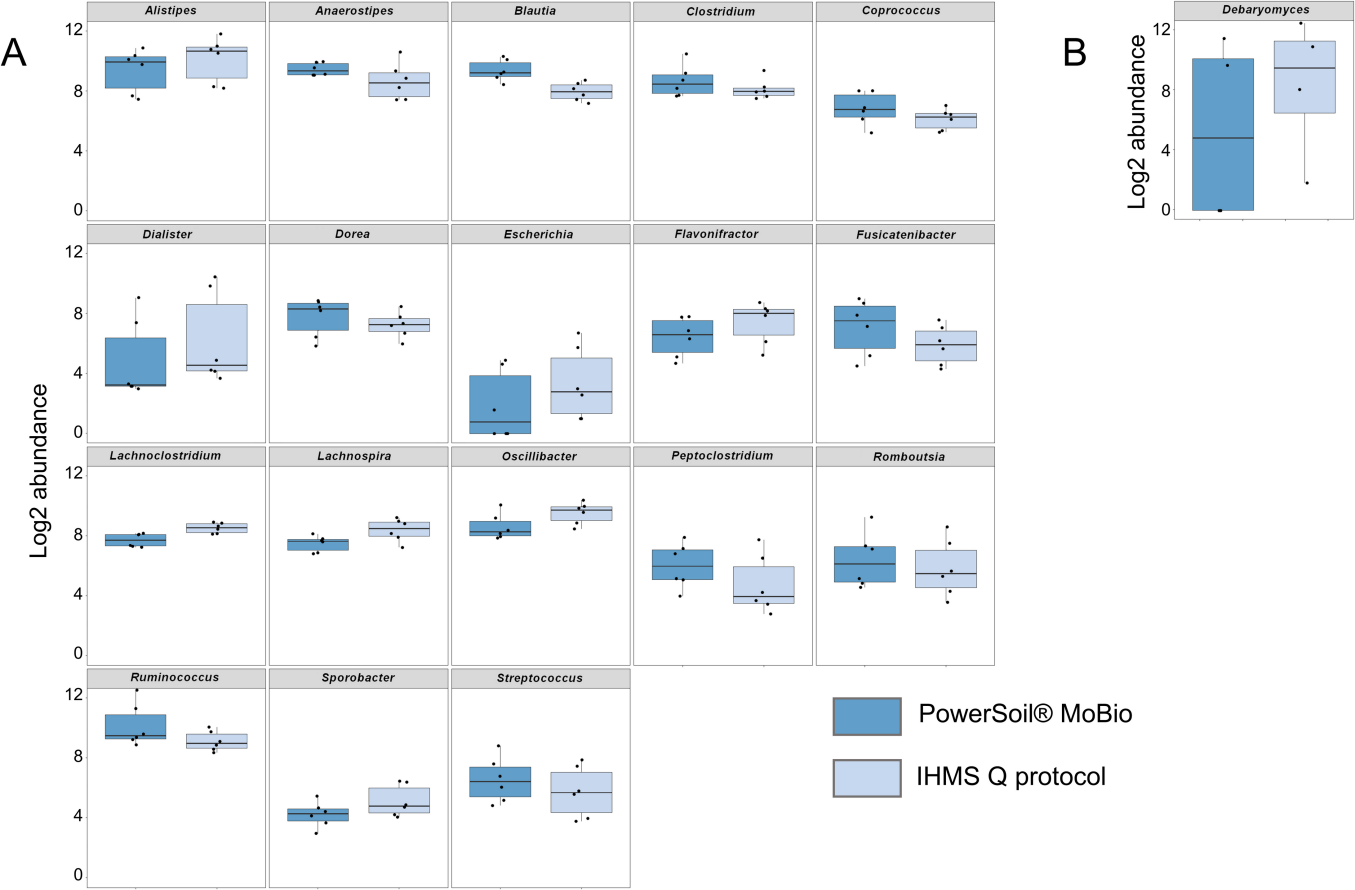
**Boxplot comparison of log2-abundance of bacterial (A) and fungal (B) taxa according to extraction protocol.** Bacterial diversity was assessed at genus level using 16S rRNA gene ultra-deep sequencing (454 technology) and fungal diversity at genus or section level using ITS1 ultra-deep sequencing. Boxplot of log2-abundance of taxa significantly different (*P*-value < 0,05) according to extraction protocols (PowerSoil® MoBio kit [dark blue] vs. IHMS Protocol Q [light blue]) during analysis at general level are presented.

Using either the IHMS Protocol Q or the PowerSoil® MoBio kit for DNA extraction, we were able to assess successfully all the taxonomic diversity (*i*.*e*. all genera/section) for both bacterial and fungal analysis (Fig 5, S5 Fig). However, the comparison at general level of relative abundances of bacterial and fungal taxa revealed significant differences for 18/41 bacterial genera (*Alistipes*, *Anaerostipes*, *Blautia*, *Clostridium*, *Coprococcus*, *Dialister*, *Dorea*, *Escherichia*, *Flavonifractor*, *Fusicatenibacter*, *Lachnoclostridium*, *Lachnospira*, *Oscillibacter*, *Peptoclostridium*, *Romboutsia*, *Ruminococcus*, *Sporobacter*, *Streptococcus*) but only 1/40 fungal (*Debaryomyces*) genera (Fig 5, S5 Table). Of note, 7/18 or these bacterial taxa were “core” taxa. Analysis at individual level showed, respectively, 2 bacterial (*Roseburia* and *Sutterella*) and 3 fungal (*Malassezia*, *Penicillium* and *Pleurotus*) additional taxa statistically different according to the extraction protocol (S5 Fig, S6 Table). Among all those taxa impacted at general or individual level by the extraction protocol, we observed higher relative abundance associated with IHMS Protocol Q in half (10/20) cases for bacteria and 3/4 for fungi (Fig 5, S5 Fig).

## Discussion

In the present study, we compared, simultaneously and under the same conditions, the impact of fecal samples storage with or without RNA*later*® and of two well-known extraction protocols (IHMS Protocol Q [39] and PowerSoil MoBio®[5]) on the concomitant assessment of bacterial and fungal intestinal microbiota composition through targeted metagenomic analyses. Although our study included a small number of subjects, our results suggest that the methodological issues associated with storage or extraction conditions might be different for bacterial or fungal metagenomic analyses. This may be of interest for authors interested in the emerging field of combined analyses.

First, we observed that the extraction protocol had an influence on the rate of double-strand DNA production, with the IHMS Protocol Q allowing the recovery of higher DNA yields. We also observed variations in relative abundance associated with extraction protocols for a large number of bacterial genera (18/41 at general level analysis). However, both extraction protocols allowed a full assessment of the bacterial taxonomic diversity (*i*.*e*., all taxa were detected by both methods). Although the number of subjects in our study was low, our results were in agreement with that of other authors, who highlighted the impact of extraction protocols on the assessment of bacterial microbiota [8,12,15,17,18]. Most of them showed, as we did, differences in yield of extracted DNA and above all significant variations in bacterial community composition. By contrast, the impact of extraction protocols on fungal microbiota appeared to be moderate in our study, with only one fungal taxon impacted during general level analysis. This result might be limited by the low number of samples analyzed, especially after the dismissal of i2 samples due to unsatisfactory rarefaction curves. However, this result is consistent with that of Huseyin *et al*, who compared 5 extraction protocols for the assessment of intestinal mycobiota [16]. In their work, they showed that the critical point associated with fungal extraction was the presence or the absence of bead-beating steps. Extraction protocols without bead-beating produced significantly lower DNA yields, resulting in difficulties or inability to amplify fungal ITS DNA. Using one or another extraction protocol associated with single or repeat bead-beating steps, they did not observe significant differences in terms of fungal diversity detected. In our study, the two extraction protocols used were both preceded by repeat bead-beating steps. Our results, combined with those of Huseyin *et al* [16], suggest that the extraction protocol might not be critical for the assessment of the fungal diversity, provided that the yield of DNA extracted is important enough to allow amplification of fungal genes, belonging to the “rare biosphere”[20]. Overall, to perform combined bacterial and fungal microbiota analyses, the choice of the extraction protocol should probably favor a method validated for an optimal assessment of the bacterial microbiota (such as the IHMS Protocol Q, which will also allow further comparison of data generated in-between studies [8,12]) that includes a powerful step of mechanical lysis (repeat bead-beating) to ensure a high DNA yield for fungal microbiota analysis.

Secondly, we observed that the storage of fecal samples after dilution in RNA*later*® prior -80°C freezing did not impact the yield of DNA extracted but that it might induce a decrease of relative abundance of some bacterial and fungal taxa (11/41 and 5/40, respectively, combining general and individual analyses). However, for 1/41 bacterial and 1/40 fungal taxa, storage with RNA*later*® allowed the detection of a higher amount of reads (higher relative abundance) compared to immediate freezing. Overall, the bacterial and fungal taxonomic diversity was fully assessed whatever storage condition was used, except for the fungal *Cryptococcus* taxon, which was detected only in samples stored in RNA*later*®. These result are of interest, as few studies have focused on the impact of RNA*later*® on bacterial diversity [6,7,11,13,14] and, to our knowledge, none on bacterial and fungal diversity together. Contrary to Dominianni *et al* [7] and Gorzelak *et al* [11], we did not observe a decrease in DNA purity or DNA yield for samples stored with RNA*later*®. This might be related to the washing pre-process (two-time centrifugation-PBS rinsing) used in our study for samples stored in RNA*later*®. Regarding the impact of RNA*later*® on diversity and relative abundance of bacterial taxa, Dominianni *et al* [7] observed, a trend towards lower bacterial diversity at phylum level in feces stored with RNA*later*® at room temperature compared to feces frozen without additive at -80°C. Sinha *et al* [6] and Gorzalek *et al* [11] showed that although RNA*later*® was good at stabilizing the microbiota across time, it might induce a lower detection of certain phyla such as *Bacteroidetes* [6] or *Firmicutes* [6,11] compared to samples processed without RNA*later*®. In our study, we conducted the analysis at the genus level and we observed an association between RNA*later*® use and a decrease in relative abundance of a moderate number of bacterial genera, some of which being “core” taxa. Overall, our results should probably warn us on possible biases in relative abundance assessment of some bacterial and fungal taxa when samples are stored after dilution in RNA*later*® and encourage us, when feasible, to prefer immediate freezing of feces without additive. However, when environmental conditions are unfavorable or when combined transcriptomic analysis is planned, RNA*later*® remains an interesting option. To confirm our findings, complementary data regarding the role of nucleic acid stabilizer solutions on combined fungal and bacterial microbiota analysis should be necessary.

Our study also emphasizes specific difficulties associated with the analysis of the fungal microbiota compared to the bacterial one. While fecal samples of healthy adults were processed exactly the same way and a similar amount of reads were obtained for 16S and ITS1 high-throughput sequencing, the fungal analysis could not be completed for one of the 3 individuals. Indeed, only a very low rate of ITS reads were assigned as fungal reads despite the use of multiple sequence databases, which lead to an insufficient assessment of the fungal diversity and the exclusion of the individual for further analysis. By comparison, all 16S reads of the same individual were fully assigned as bacterial reads. This fact, also described by other authors [16,37,50], raises two hypotheses: it might either be a problem of unspecific DNA amplification due to the very low yield of fungal DNA obtained from fecal samples extraction (“rare biosphere” [20]) or a problem of insufficient annotation due to incomplete ITS sequence databases. This last issue was already discussed by different authors [37,46,50,51], who highlighted the high rate of incomplete, incorrect or redundant taxonomic assignment in public fungal sequence databases. They also emphasize the evolving nature of the phylogenic relationships between fungi and the heterogeneity of fungal taxonomy to explain the difficulty to build a well-established and commonly-accepted database. In 2015, Tang *et al* [37], after comparing the results of three existing databases (UNITE [45], Findley [46], and RefSeq Targeted Loci [42] databases), observed that the distribution of fungi detected in a sample could vary significantly according to the database used and thus proposed a new hand-curated database (the THF database). Unfortunately, two years later, using a combination of the most notorious ITS databases, we had to face the same difficulty again, as did Huseyin *et al* [16], who obtained extremely high percentages of unassigned ITS reads (> 90%) for 2/18 subjects. The amplification and/or database challenge is still a crucial limitation for ITS targeted metagenomics analysis, leading to underestimation of mycobiota diversity, and these issues require particular attention from the mycologist community over the upcoming years.

Overall, and as described previously [6,7,18], we observed important inter-individual variability of the intestinal microbiota in our study. This phenomenon was particularly visible for the fungal microbiota. Over three subjects, one (i2) had to be withheld from the study because most of her ITS reads could not be assigned, suggesting she was harboring yet unknown or rare fungal sequences; and the two other did not share a common “core” of fungal genera, except for the predominant *Penicillium* taxon. This huge variability of the fungal intestinal microbiota assessed through metagenomics has already been highlighted by previous authors [16,21,37]. In their work on 16 healthy adults following a vegetarian diet, Suhr *et al* [37] observed that no taxon (analyzed at genus level) was common to all individuals and that even samples collected from the same individual at different time showed little similarity. Huseyin *et al* [16] observed the same variability in the intestinal mycobiota of 18 healthy adults, and pointed out the importance of fungal sequences of dietary origin retrieved in fecal samples. In our study, one subject (i1) presented a very diversified intestinal mycobiota profile (with 12 genera or section highly represented) while the other (i3) had a low diversified profile (only 3 predominant genera). As *Galactomyces* and *Penicillium* were particularly over-represented in this subject, one might hypothesize that her mycobiota was submitted to an important load of fungi of dietary origin, such as French cheese, known to harbour high loads of molds, and especially *Galactomyces* and *Penicillium* species [52].

## Conclusion

Although our study included a small number of subjects, our results might provide interesting information for authors implicated in the field of combined bacterial and fungal intestinal microbiota analysis. First, we observed that methods of DNA extraction influence the yield of DNA extracted, which is highly important for fungal analysis, as the fungal DNA is part of the “rare biosphere” in the intestinal microbiota. Our results also suggested, as previously described by others, that DNA extraction influence the structure of bacterial communities in terms of relative abundance of major and minor taxa. In contrast, and taking into account the size limitation of our study, methods of DNA extraction appeared to have a lower impact on fungal communities. To perform combined bacterial and fungal microbiota analyses, we suggest that the choice of the extraction protocol should favor a method (i) validated for an optimal assessment of the bacterial microbiota, such as the IHMS Protocol Q and (ii) including repeated bead-beating steps, a procedure essential to obtain high yields of DNA allowing the amplification of fungal genes. Compared to the critical issue of extraction protocols, our results suggested the use of acid nucleic stabilizer solutions (such as RNA*later*®) for samples storage has a lower impact on bacterial and fungal community structures. However, researchers should be aware of the possible biases (such as the reduction of the detection of some bacterial and fungal genera) associated with nucleic acid stabilizer solutions, and thus use them cautiously, only when necessary.

## Supporting information

S1 Fig Comparison of DNA extracted from fecal samples stored in two different conditions using two extraction protocols. Three fecal samples from healthy individuals stored at -80°C after dilution in RNA*later*® or without additive, were submitted to 2 extraction protocols: the IHMS Protocol Q and the PowerSoil® MoBio kit.
(TIF)

S2 Fig **Rarefaction curves of bacterial (A) and fungal (B) diversity assessed from fecal samples using 16S or ITS1 ultra-deep sequencing (454 technology).** Total DNA was extracted from fecal samples of 3 healthy individuals (i1, i2 and i3) using two storage conditions (within two-hours freezing or RNA*later*® dilution before freezing) and two extraction protocols (IHMS Protocol Q and PowerSoil® MoBio kit). Bacterial diversity was assessed at genus level; fungal diversity at genus or section level.
IHMS1 = i1, RNA*later*®, IHMS Protocol Q; IHMS2 = i1, no RNA*later*®, IHMS Protocol Q;
MOBIO1 = i1, RNA*later*®, PowerSoil® MoBio kit; MOBIO2 = i1, no RNA*later*®, PowerSoil® MoBio kit;
IHMS3 = i2, RNA*later*®, IHMS Protocol Q; IHMS4 = i2, no RNA*later*®, IHMS Protocol Q;
MOBIO3 = i2, RNA*later*®, PowerSoil® MoBio kit; MOBIO4 = i2, no RNA*later*®, PowerSoil® MoBio kit;
IHMS5 = i3, RNA*later*®, IHMS Protocol Q; IHMS6 = i3, no RNA*later*®, IHMS Protocol Q;
MOBIO5 = i3, RNA*later*®, PowerSoil® MoBio kit; MOBIO6 = i3, no RNA*later*®, PowerSoil® MoBio kit.
(TIF)

S3 Fig **PCoA plots of bacterial (A) and fungal (B) between-sample dissimilarities according to storage or extraction condition.** PCoA plots, computed using bray distance, of fecal samples of 3 healthy individuals (i1 [blue], i2 [green] and i3 [orange]) processed using two storage conditions (within two-hours freezing [triangle] or RNA*later*® dilution before freezing [square]) and two extraction protocols (IHMS Protocol Q [dark color] and PowerSoil® MoBio kit [light color]) are presented. Sixty-five percent and 82% of between-sample variations were explained by the first two PC1 and PC2 axis for bacterial and fungal PCoA analyses, respectively.
(TIF)

S4 Fig **Boxplot comparison, at individual level, of log2-abundance of bacterial (A) and fungal (B) taxa according to storage condition.** Bacterial diversity was assessed at genus level using 16S rRNA gene ultra-deep sequencing (454 technology) and fungal diversity at genus or section level using ITS1 ultra-deep sequencing. Boxplot of log2-abundance of taxa significantly different (*P*-value < 0,05 at general or individual level) according to storage condition (RNA*later*® dilution before freezing [dark colors] vs. within two-hours freezing without additive [light colors]) are presented separately for each individual (i1 [blue], i2 [green], i3 [orange]). Significant differences observed at individual level are indicated using colored asterisks placed above/below boxplots. Significant differences observed at general level are indicated using black asterisks attached to the genera/sections names.
(TIF)

S5 Fig **Boxplot comparison, at individual level, of log2-abundance of bacterial (A) and fungal (B) taxa according to extraction protocol.** Bacterial diversity was assessed at genus level using 16S rRNA gene ultra-deep sequencing (454 technology) and fungal diversity at genus or section level using ITS1 ultra-deep sequencing. Boxplot of log2-abundance of taxa significantly different (*P*-value < 0,05 at general or individual level) according to extraction protocol (PowerSoil® MoBio kit [dark colors] vs. IHMS Protocol Q [light colors]) are presented separately for each individual (i1 [blue], i2 [green], i3 [orange]). Significant differences observed at individual level are indicated using colored asterisks placed above/below boxplots. Significant differences observed at general level are indicated using black asterisks attached to the genera/sections names.
(TIF)

S1 Table Results of ultra-deep sequencing (reads, OTUs and assignment) for 16S and ITS1 targets (12 samples). (DOCX)

S2 Table Alpha diversity measurements for bacterial (A) and fungal (B) metagenomic analyzes of 3 fecal samples using 2 storage and 2 extraction conditions.
(DOCX)

S3 Table Abundance Fold Change of bacterial (A) and fungal (B) taxa significantly different according to storage condition at general level.
(DOCX)

S4 Table Abundance Fold Change of bacterial (A) and fungal (B) taxa significantly different according to storage condition at individual level.
(DOCX)

S5 Table Abundance Fold Change of bacterial (A) and fungal (B) taxa significantly different according to extraction protocol at general level.
(DOCX)

S6 Table Abundance Fold Change of bacterial (A) and fungal (B) taxa significantly different according to extraction protocol at individual level.
(DOCX)
