# Supplementary figures and images for: Combined bacterial and fungal intestinal microbiota analyses: Impact of storage conditions and DNA extraction protocols
Source: PLoS One. 2018 Aug 3;13(8):e0201174. doi: 10.1371/journal.pone.0201174 (PMC6075747; doi:10.1371/journal.pone.0201174)

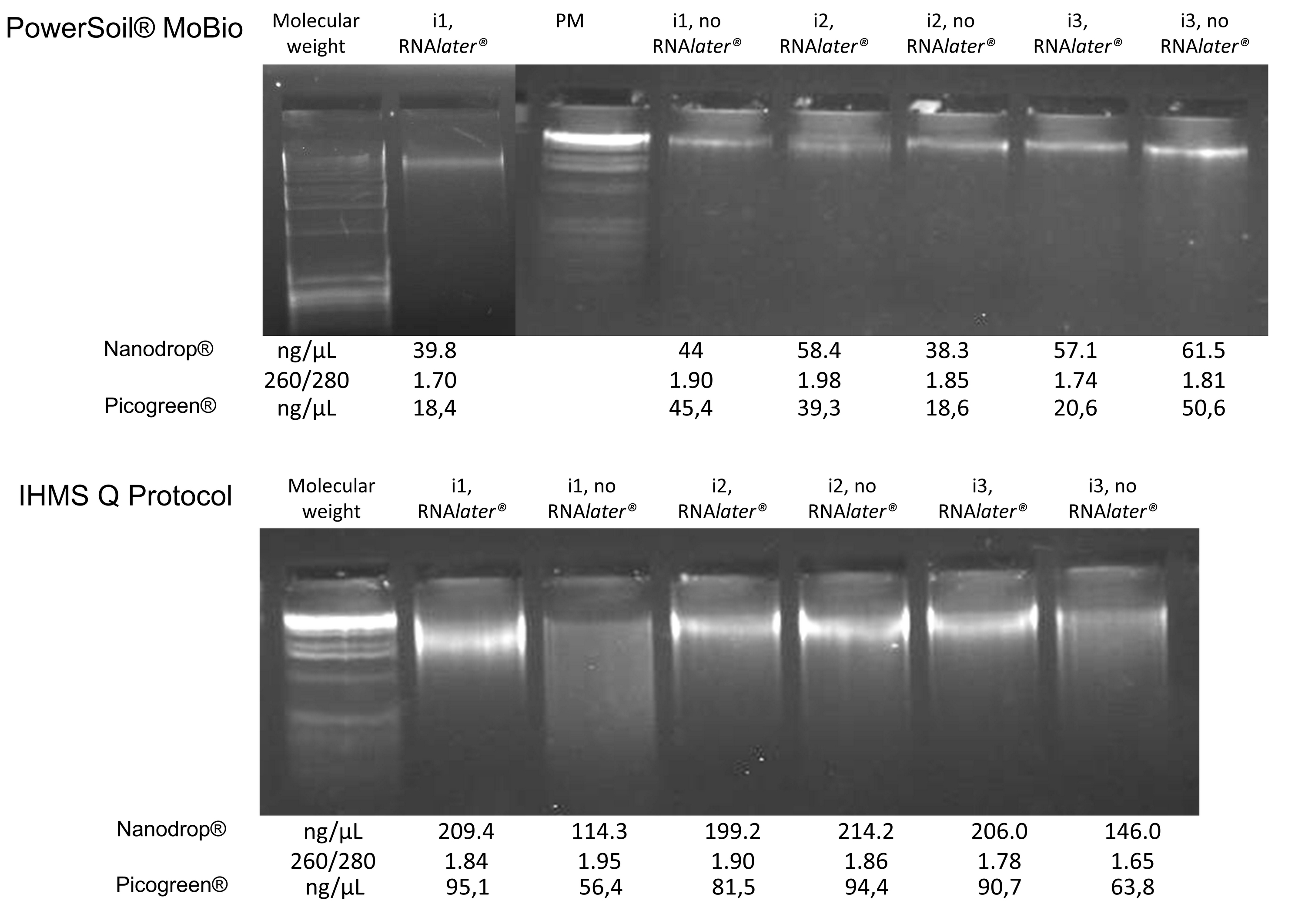

Supplement: S1 Fig — Three fecal samples from healthy individuals stored at -80°C after dilution in RNAlater® or without additive, were submitted to 2 extraction protocols: the IHMS Protocol Q and the PowerSoil® MoBio kit. (TIF) [file pone.0201174.s001.tif]

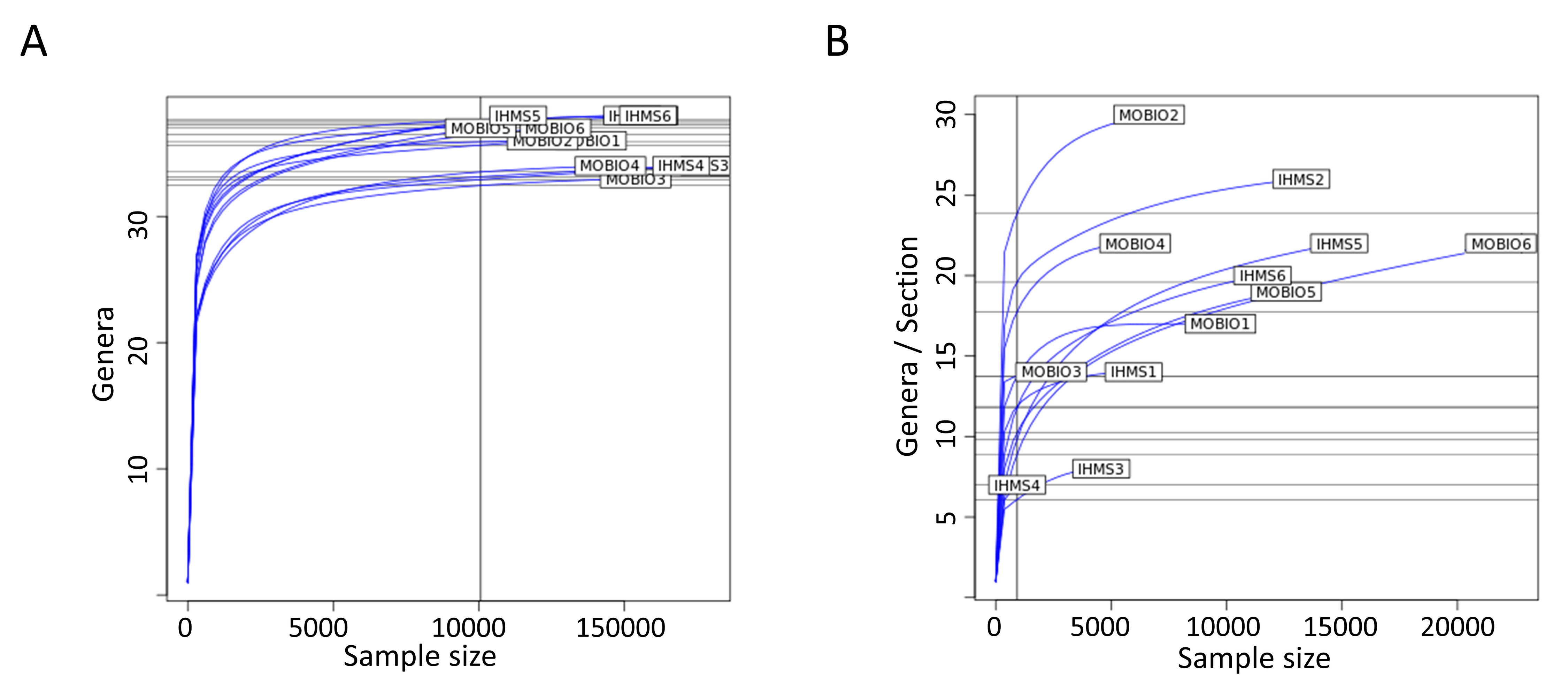

Supplement: S2 Fig — Rarefaction curves of bacterial (A) and fungal (B) diversity assessed from fecal samples using 16S or ITS1 ultra-deep sequencing (454 technology). Total DNA was extracted from fecal samples of 3 healthy individuals (i1, i2 and i3) using two storage conditions (within two-hours freezing or RNAlater® dilution before freezing) and two extraction protocols (IHMS Protocol Q and PowerSoil® MoBio kit). Bacterial diversity was assessed at genus level; fungal diversity at genus or section level. IHMS1 = i1, RNAlater®, IHMS Protocol Q; IHMS2 = i1, no RNAlater®, IHMS Protocol Q; MOBIO1 = i1, RNAlater®, PowerSoil® MoBio kit; MOBIO2 = i1, no RNAlater®, PowerSoil® MoBio kit; IHMS3 = i2, RNAlater®, IHMS Protocol Q; IHMS4 = i2, no RNAlater®, IHMS Protocol Q; MOBIO3 = i2, RNAlater®, PowerSoil® MoBio kit; MOBIO4 = i2, no RNAlater®, PowerSoil® MoBio kit; IHMS5 = i3, RNAlater®, IHMS Protocol Q; IHMS6 = i3, no RNAlater®, IHMS Protocol Q; MOBIO5 = i3, RNAlater®, PowerSoil® MoBio kit; MOBIO6 = i3, no RNAlater®, PowerSoil® MoBio kit. (TIF) [file pone.0201174.s002.tif]

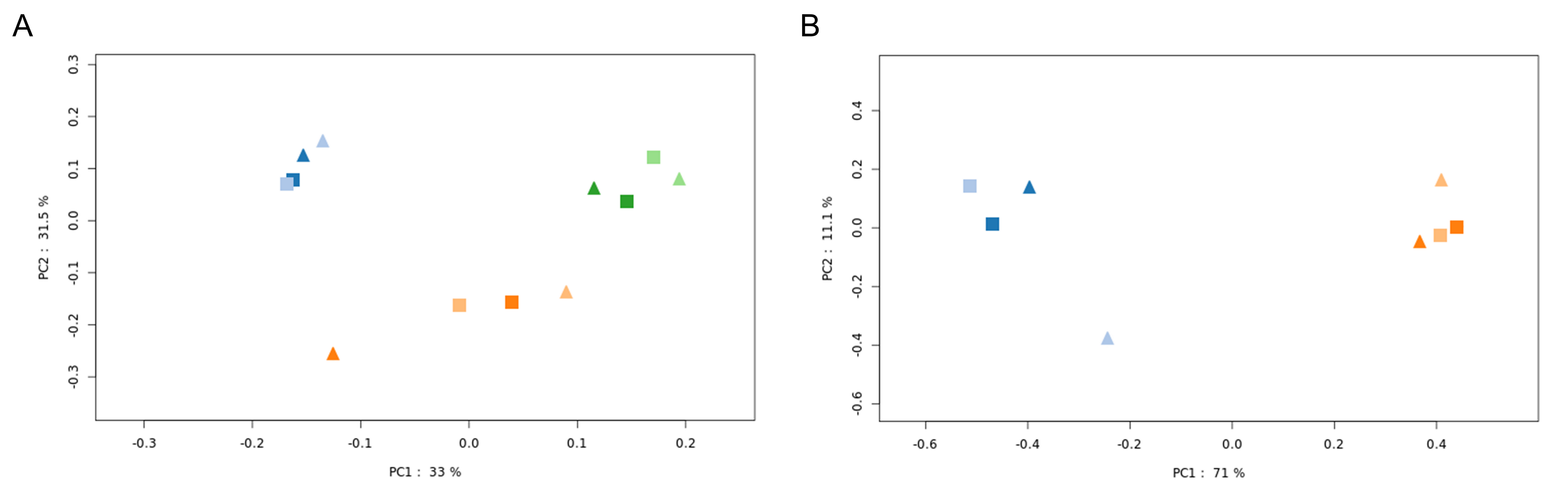

Supplement: S3 Fig — PCoA plots of bacterial (A) and fungal (B) between-sample dissimilarities according to storage or extraction condition. PCoA plots, computed using bray distance, of fecal samples of 3 healthy individuals (i1 [blue], i2 [green] and i3 [orange]) processed using two storage conditions (within two-hours freezing [triangle] or RNAlater® dilution before freezing [square]) and two extraction protocols (IHMS Protocol Q [dark color] and PowerSoil® MoBio kit [light color]) are presented. Sixty-five percent and 82% of between-sample variations were explained by the first two PC1 and PC2 axis for bacterial and fungal PCoA analyses, respectively. (TIF) [file pone.0201174.s003.tif]

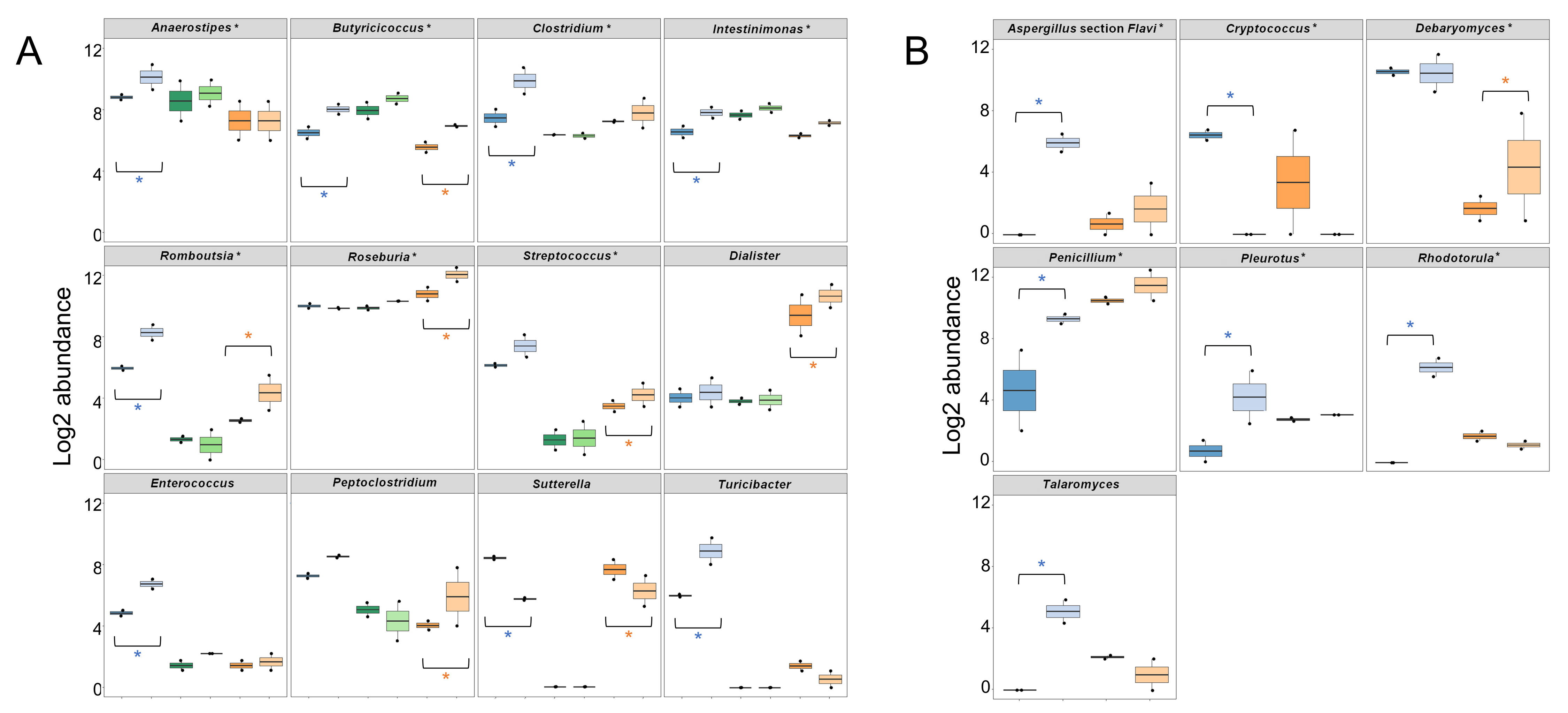

Supplement: S4 Fig — Boxplot comparison, at individual level, of log2-abundance of bacterial (A) and fungal (B) taxa according to storage condition. Bacterial diversity was assessed at genus level using 16S rRNA gene ultra-deep sequencing (454 technology) and fungal diversity at genus or section level using ITS1 ultra-deep sequencing. Boxplot of log2-abundance of taxa significantly different (P-value < 0,05 at general or individual level) according to storage condition (RNAlater® dilution before freezing [dark colors] vs. within two-hours freezing without additive [light colors]) are presented separately for each individual (i1 [blue], i2 [green], i3 [orange]). Significant differences observed at individual level are indicated using colored asterisks placed above/below boxplots. Significant differences observed at general level are indicated using black asterisks attached to the genera/sections names. (TIF) [file pone.0201174.s004.tif]

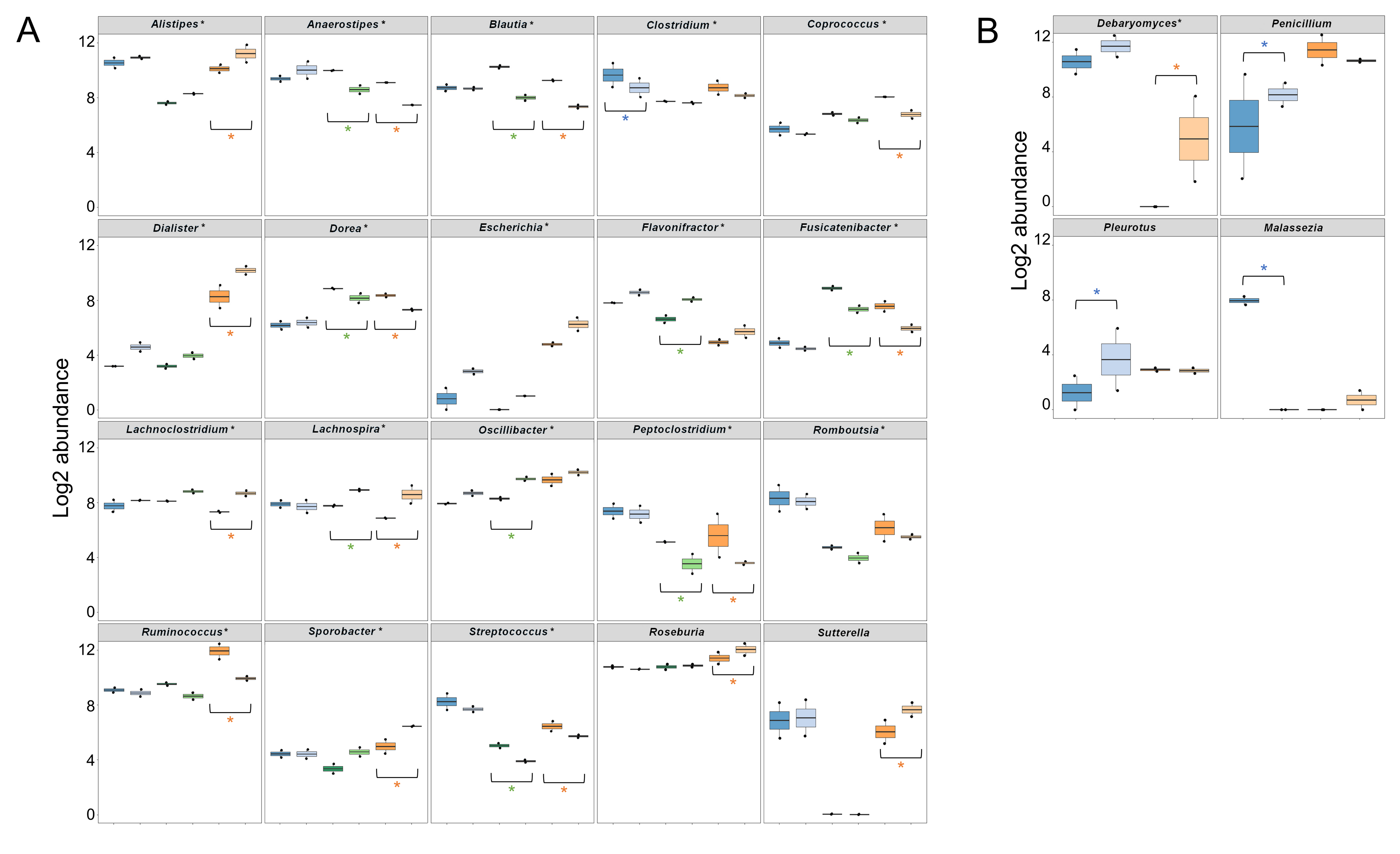

Supplement: S5 Fig — Boxplot comparison, at individual level, of log2-abundance of bacterial (A) and fungal (B) taxa according to extraction protocol. Bacterial diversity was assessed at genus level using 16S rRNA gene ultra-deep sequencing (454 technology) and fungal diversity at genus or section level using ITS1 ultra-deep sequencing. Boxplot of log2-abundance of taxa significantly different (P-value < 0,05 at general or individual level) according to extraction protocol (PowerSoil® MoBio kit [dark colors] vs. IHMS Protocol Q [light colors]) are presented separately for each individual (i1 [blue], i2 [green], i3 [orange]). Significant differences observed at individual level are indicated using colored asterisks placed above/below boxplots. Significant differences observed at general level are indicated using black asterisks attached to the genera/sections names. (TIF) [file pone.0201174.s005.tif]
