# Supplementary material for: Combined bacterial and fungal intestinal microbiota analyses: Impact of storage conditions and DNA extraction protocols
Source: PLoS One. 2018 Aug 3;13(8):e0201174. doi: 10.1371/journal.pone.0201174 (PMC6075747; doi:10.1371/journal.pone.0201174)
Supplement: S1 Table — (DOCX) [file pone.0201174.s006.docx]

**S1 Table.** Results of the high-throughput sequencing (reads, OTUs and assignment) of bacterial V3-V4 16S and fungal ITS1 sequencing of 12 samples (n=3 individuals i1, i2 and i3 X n=2 storage conditions X n=2 extraction protocols).

|  | **Overall** | | | **i1** | | **i2** | | **i3** | |
| --- | --- | --- | --- | --- | --- | --- | --- | --- | --- |
|  | Reads *(assigned %)* | OTUs | Reads *(assigned %)* | | OTUs | Reads *(assigned %)* | OTUs | Reads *(assigned %)* | OTUs |
| **V3-V4 16S reads after trimming** | **171869** | **130** | **57188** | | **111** | **64803** | **102** | **49878** | **125** |
| Assigned as Bacteria | 171869 *(100)* | 130 | 57188 *(100)* | | 111 | 64803 *(100)* | 102 | 49878 *(100)* | 125 |
| At species level | 118527 *(69.0)* | 52 | 40133 *(70.2)* | | 47 | 45813 *(70.7)* | 42 | 32581 *(65.3)* | 51 |
| At genus level | 52943 *(30.8)* | 74 | 16721 *(29.2)* | | 60 | 18956 *(29.3)* | 58 | 17266 *(34.6)* | 71 |
| Unidentified bacteria | 399 *(0.2)* | 4 | 334 *(0.6)* | | 4 | 34 *(0.05)* | 2 | 31 *(0.1)* | 3 |
| Non-assigned reads | 0 *(0)* | 0 | 0 *(0)* | | 00 | 0 *(0)* | 0 | 0 *(0)* | 0 |
| **ITS1 reads after trimming** | **199089** | **272** | **66598** | | **164** | **62238** | **149** | **70253** | **120** |
| Assigned as Fungi | 102478 *(51.5)* | 81 | 29313 *(44.0)* | | 60 | 7924 *(12.7)* | 39 | 65241 *(92.9)* | 52 |
| At species level | 66935 *(*33.6*)* | 51 | 8589 *(12.9)* | | 39 | 5193 *(8.3)* | 26 | 53144 *(75.6)* | 32 |
| At genus/section level | 35373 *(*17.8*)* | 29 | 20545 *(30.8)* | | 20 | 2731 *(4.4)* | 13 | 12097 *(1702)* | 20 |
| Unidentified fungi | 170 *(0.1)* | 1 | 170 *(0.3)* | | 1 | 0 *(0)* | 0 | 0 *(0)* | 0 |
| Assigned as Plantae | 2434 *(1.2)* | 15 | 1676 *(2.5)* | | 13 | 667 *(2.5)* | 6 | 91 *(0.1)* | 5 |
| Non-assigned reads | 94177 *(*47.3*)* | 176 | 35609 *(53.5)* | | 91 | 53647 *(53.5)* | 104 | 4921 *(7.0)* | 63 |

Footnote: All reads are available on EBI ENA (Accession number PRJEB25216; https://www.ebi.ac.uk/ena).

|  |
| --- |
