# Supplementary material for: Combined bacterial and fungal intestinal microbiota analyses: Impact of storage conditions and DNA extraction protocols
Source: PLoS One. 2018 Aug 3;13(8):e0201174. doi: 10.1371/journal.pone.0201174 (PMC6075747; doi:10.1371/journal.pone.0201174)
Supplement: S2 Table — Alpha diversity measurements for bacterial (A) and fungal (B) metagenomic analyzes of 3 fecal samples using 2 storage and 2 extraction conditions. (DOCX) [file pone.0201174.s007.docx]

**S2 Table.** Alpha diversity measurements (Shannon, Simpson indexes) for bacterial (A) and fungal (B) metagenomic analyzes (454 technology) performed on fecal samples of 3 healthy individuals (i1, i2 and i3) using two storage conditions (within two hours freezing or RNA*later*® storage) and two extraction protocols (IHMS Protocol Q and PowerSoil® MoBio kit).

| **A - Bacterial diversity** | **Shannon** | **Simpson** |
| --- | --- | --- |
| MOBIO-RNALater | 2.48 ± 0.15 | 0.86 ± 0.03 |
| MOBIO-Wihtin-2h freezing | 2.58± 0.16 | 0.88 ± 0.02 |
| IHMS-RNALater | 2.54 ± 0.05 | 0.87 ± 0.00 |
| IHMS-Wihtin-2h freezing | 2.60 ± 0.14 | 0.89 ± 0.01 |
| **B - Fungal diversity** | **Shannon** | **Simpson** |
| MOBIO-RNALater | 1.14 ± 0.28 | 0.52 ± 0.05 |
| MOBIO-Wihtin-2h freezing | 1.51 ± 1.08 | 0.64 ± 0.29 |
| IHMS-RNALater | 1.10 ± 0.00 | 0.54 ± 0.09 |
| IHMS-Wihtin-2h freezing | 1.15 ± 0.12 | 0.52 ± 0.16 |
