# Supplementary material for: Combined bacterial and fungal intestinal microbiota analyses: Impact of storage conditions and DNA extraction protocols
Source: PLoS One. 2018 Aug 3;13(8):e0201174. doi: 10.1371/journal.pone.0201174 (PMC6075747; doi:10.1371/journal.pone.0201174)
Supplement: S3 Table — Abundance Fold Change of bacterial (A) and fungal (B) taxa significantly different according to storage condition at general level. (DOCX) [file pone.0201174.s008.docx]

**Table S3.** Table presenting the Fold Change (expressed as log2 FoldChange) between abundances of bacterial (A) and fungal (B) genera significantly different (*P*-value < 0,05) according to the storage condition (RNA*later*® dilution before freezing or within two-hours freezing) at general level.

| **A - Bacterial genera** | **baseMean** | **log2 FoldChange** | ***P-*value** |
| --- | --- | --- | --- |
| *Butyricicoccus* | 352 | 0.69 | <0.001 |
| *Intestinimonas* | 325 | 0.47 | <0.001 |
| *Romboutsia* | 142 | 0.70 | 0.001 |
| *Clostridium* | 428 | 0.53 | 0.005 |
| *Roseburia* | 1433 | 0.29 | 0.005 |
| *Anaerostipes* | 657 | 0.34 | 0.039 |
| *Streptococcus* | 121 | 0.39 | 0.049 |
| **B - Fungal genera/section** | **baseMean** | **log2 FoldChange** | ***P*-value** |
| *Penicillium* | 4194 | 1.59 | <0.001 |
| *Cryptococcus* | 66 | -4.75 | 0.005 |
| *Aspergillus* section *Flavi* | 31.75 | 3.16 | 0.03 |
| *Debaryomyces* | 2507 | 0.98 | 0.037 |
| *Pleurotus* | 18 | 1.59 | 0.037 |
| *Rhodotorula* | 37 | 2.61 | 0.037 |
