# Supplementary material for: Combined bacterial and fungal intestinal microbiota analyses: Impact of storage conditions and DNA extraction protocols
Source: PLoS One. 2018 Aug 3;13(8):e0201174. doi: 10.1371/journal.pone.0201174 (PMC6075747; doi:10.1371/journal.pone.0201174)
Supplement: S4 Table — Abundance Fold Change of bacterial (A) and fungal (B) taxa significantly different according to storage condition at individual level. (DOCX) [file pone.0201174.s009.docx]

**Table S4.** Table presenting the Fold Change (expressed as log2 FoldChange) between abundances of bacterial (A) and fungal (B) genera significantly different (*P*-value < 0,05) according to the storage condition (RNA*later*® dilution before freezing or within two-hours freezing) at individual level.

| **A - Bacterial genera** | **baseMean** | **log2 FoldChange** | ***P*-value** |
| --- | --- | --- | --- |
| **i1** |  |  |  |
| *Sutterella* | 74 | -2.49 | <0.001 |
| *Romboutsia** | 142 | 1.52 | <0.001 |
| *Clostridium** | 428 | 1.57 | <0.001 |
| *Turicibacter* | 62 | 2.67 | <0.001 |
| *Intestinimonas** | 324 | 0.81 | 0.002 |
| *Butyricicoccus** | 352 | 0.99 | 0.002 |
| *Anaerostipes** | 657 | 0.85 | 0.007 |
| *Streptococcus** | 121 | 0.82 | 0.009 |
| *Enterococcus* | 17 | 1.79 | 0.046 |
| **i2^a^** |  |  |  |
| **i3** |  |  |  |
| *Roseburia** | 1433 | 0.82 | <0.001 |
| *Butyricicoccus** | 352 | 0.90 | 0.014 |
| *Romboutsia** | 142 | 1.17 | 0.014 |
| *Sutterella* | 74 | -1.26 | 0.014 |
| *Peptoclostridium* | 78 | 1.85 | 0.046 |
| *Dialister* | 265 | 1.13 | 0.049 |
| **B - Fungal genera/section** | **baseMean** | **log2 FoldChange** | ***P*-value** |
| **i1** |  |  |  |
| *Penicillium** | 4194 | 4.45 | <0.001 |
| *Aspergillus* section *Flavi** | 32 | 9.25 | 0.020 |
| *Pleurotus** | 18 | 5.10 | 0.020 |
| *Rhodotorula** | 37 | 9.77 | 0.020 |
| *Talaromyces* | 18 | 8.99 | 0.020 |
| *Cryptococcus** | 66 | -9.80 | 0.037 |
| **i3** |  |  |  |
| *Debaryomyces** | 2507 | 3.54 | 0.015 |

Footnote:

* Genera found with significant log2 abundance differences during analysis at individual and general level.

^a^ No significant abundance differences for bacterial genera was observed for i2 comparing storage condition with or without RNA*later*®*.*
