# Supplementary material for: Combined bacterial and fungal intestinal microbiota analyses: Impact of storage conditions and DNA extraction protocols
Source: PLoS One. 2018 Aug 3;13(8):e0201174. doi: 10.1371/journal.pone.0201174 (PMC6075747; doi:10.1371/journal.pone.0201174)
Supplement: S5 Table — Abundance Fold Change of bacterial (A) and fungal (B) taxa significantly different according to extraction protocol at general level. (DOCX) [file pone.0201174.s010.docx]

**Table S5.** Table presenting the Fold Change (expressed as log2 FoldChange) between abundances of bacterial (A) and fungal (B) genera significantly different (*P*-value < 0,05) according to the extraction protocol (PowerSoil® MoBio kit or IHMS Protocol Q) at general level.

| **A - Bacterial genera** | **baseMean** | **log2 FoldChange** | ***P*-value** |
| --- | --- | --- | --- |
| *Lachnospira* | 284 | 0.78 | <0.001 |
| *Dialister* | 265 | 1.20 | <0.001 |
| *Lachnoclostridium* | 302 | 0.70 | <0.001 |
| *Streptococcus* | 121 | -0.71 | <0.001 |
| *Oscillibacter* | 644 | 0.79 | <0.001 |
| *Anaerostipes* | 657 | -0.69 | <0.001 |
| *Flavonifractor* | 175 | 0.87 | <0.001 |
| *Fusicatenibacter* | 156 | -1.03 | <0.001 |
| *Blautia* | 513 | -1.21 | <0.001 |
| *Ruminococcus* | 1254 | -0.89 | 0.001 |
| *Peptoclostridium* | 78 | -1.17 | 0.001 |
| *Dorea* | 231 | -0.44 | 0.002 |
| *Sporobacter* | 32 | 0.81 | 0.003 |
| *Coprococcus* | 111 | -0.61 | 0.010 |
| *Clostridium* | 428 | -0.47 | 0.010 |
| *Alistipes* | 1293 | 0.62 | 0.010 |
| *Romboutsia* | 142 | -0.50 | 0.012 |
| *Escherichia* | 19 | 2.69 | 0.026 |
| **B - Fungal genera/section** | **baseMean** | **log2 FoldChange** | ***P*-value** |
| *Debaryomyces* | 2507 | 1.74 | <0.001 |
