# Supplementary material for: Combined bacterial and fungal intestinal microbiota analyses: Impact of storage conditions and DNA extraction protocols
Source: PLoS One. 2018 Aug 3;13(8):e0201174. doi: 10.1371/journal.pone.0201174 (PMC6075747; doi:10.1371/journal.pone.0201174)
Supplement: S6 Table — Abundance Fold Change of bacterial (A) and fungal (B) taxa significantly different according to extraction protocol at individual level. (DOCX) [file pone.0201174.s011.docx]

**Table S6.** Table presenting the Fold Change (expressed as log2 FoldChange) between abundances of bacterial (A) and fungal (B) genera significantly different (*P*-value < 0,05) according to the extraction protocol (PowerSoil® MoBio kit or IHMS Protocol Q) at individual level.

| **A - Bacterial genera** | **baseMean** | **log2 FoldChange** | ***P*-value** |
| --- | --- | --- | --- |
| **i1** |  |  |  |
| *Clostridium** | 428 | -0.92 | 0.044 |
| **i2** |  |  |  |
| *Blautia** | 513 | -2.26 | <0.001 |
| *Fusicatenibacter** | 156 | -1.54 | <0.001 |
| *Anaerostipes** | 657 | -1.40 | <0.001 |
| *Flavonifractor** | 175 | 1.45 | <0.001 |
| *Oscillibacter** | 644 | 1.46 | <0.001 |
| *Streptococcus** | 121 | -1.20 | 0.006 |
| *Lachnospira** | 284 | 1.15 | 0.013 |
| *Dorea** | 231 | -0.70 | 0.016 |
| *Peptoclostridium** | 78 | -1.64 | 0.048 |
| **i3** |  |  |  |
| *Blautia** | 513 | -1.92 | <0.001 |
| *Anaerostipes** | 657 | -1.64 | <0.001 |
| *Fusicatenibacter** | 156 | -1.62 | <0.001 |
| *Lachnospira** | 284 | 1.75 | <0.001 |
| *Ruminococcus** | 1254 | -2.01 | <0.001 |
| *Dialister** | 265 | 1.91 | <0.001 |
| *Lachnoclostridium** | 302 | 1.35 | <0.001 |
| *Dorea** | 231 | -1.05 | <0.001 |
| *Sutterella* | 74 | 1.51 | 0.001 |
| *Roseburia* | 1433 | 0.59 | 0.004 |
| *Sporobacter** | 32 | 1.51 | 0.004 |
| *Coprococcus** | 111 | -1.29 | 0.005 |
| *Peptoclostridium** | 78 | -2.23 | 0.005 |
| *Alistipes** | 1293 | 1.10 | 0.030 |
| *Streptococcus** | 121 | -0.75 | 0.049 |
| **B - Fungal genera/section** | **baseMean** | **log2 FoldChange** | ***P*-value** |
| **i1** |  |  |  |
| *Malassezia* | 131 | -12,06 | 0,003 |
| *Penicillium* | 4194 | 2,37 | 0,036 |
| *Pleurotus* | 18 | 3,68 | 0,049 |
| **i3** |  |  |  |
| *Debaryomyces** | 2507 | 4,98 | 0,001 |

Footnote:

* Genera found with significant log2 abundance differences during analysis at individual and general level.
